# Antimicrobial Resistance of Hypervirulent *Klebsiella pneumoniae*: Epidemiology, Hypervirulence-Associated Determinants, and Resistance Mechanisms

**DOI:** 10.3389/fcimb.2017.00483

**Published:** 2017-11-21

**Authors:** Chang-Ro Lee, Jung Hun Lee, Kwang Seung Park, Jeong Ho Jeon, Young Bae Kim, Chang-Jun Cha, Byeong Chul Jeong, Sang Hee Lee

**Affiliations:** ^1^National Leading Research Laboratory of Drug Resistance Proteomics, Department of Biological Sciences, Myongji University, Yongin, South Korea; ^2^Biotechnology Program, North Shore Community College, Danvers, MA, United States; ^3^Department of Systems Biotechnology, College of Biotechnology and Natural Resources, Chung-Ang University, Anseong, South Korea

**Keywords:** antimicrobial resistance, hypervirulent *Klebsiella pneumoniae*, epidemiology, resistance mechanism, serotype, sequence type

## Abstract

*Klebsiella pneumoniae* is one of the most clinically relevant species in immunocompromised individuals responsible for community-acquired and nosocomial infections, including pneumonias, urinary tract infections, bacteremias, and liver abscesses. Since the mid-1980s, hypervirulent *K. pneumoniae*, generally associated with the hypermucoviscosity phenotype, has emerged as a clinically significant pathogen responsible for serious disseminated infections, such as pyogenic liver abscesses, osteomyelitis, and endophthalmitis, in a generally younger and healthier population. Hypervirulent *K. pneumoniae* infections were primarily found in East Asia and now are increasingly being reported worldwide. Although most hypervirulent *K. pneumoniae* isolates are antibiotic-susceptible, some isolates with combined virulence and resistance, such as the carbapenem-resistant hypervirulent *K. pneumoniae* isolates, are increasingly being detected. The combination of multidrug resistance and enhanced virulence has the potential to cause the next clinical crisis. To better understand the basic biology of hypervirulent *K. pneumoniae*, this review will provide a summarization and discussion focused on epidemiology, hypervirulence-associated factors, and antibiotic resistance mechanisms of such hypervirulent strains. Epidemiological analysis of recent clinical isolates in China warns the global dissemination of hypervirulent *K. pneumoniae* strains with extensive antibiotic resistance in the near future. Therefore, an immediate response to recognize the global dissemination of this hypervirulent strain with resistance determinants is an urgent priority.

## Introduction

*Klebsiella pneumoniae* is an opportunistic pathogen in immunocompromised individuals that causes a wide range of infections, including pneumonia, urinary tract infection, bacteremia, and meningitis, such as people suffering from diabetes or malignancies (Paczosa and Mecsas, 2016). *K. pneumoniae* has a polysaccharide capsule that is important for its pathogenesis and its ability to avoid phagocytosis (Cortés et al., 2002). Over the past two decades, unlike the “classic” *K. pneumoniae* (cKP), a new “hypervirulent” *K. pneumoniae* (hvKP) with hypermucoviscosity has emerged as a clinically significant pathogen causing highly invasive infections, such as liver abscesses, in both healthy and immunocompromised individuals (Prokesch et al., 2016). Remarkably, these infections are often complicated by devastating disseminated infections, including endophthalmitis and meningitis (Shon et al., 2013). This ability to disseminate is a common feature of certain Gram-positive pathogens, such as *Staphylococcus aureus*, but is generally uncommon for enteric Gram-negative bacilli, including *K. pneumoniae*, in the absence of host susceptibility factors (Cheng et al., 1991; Shon et al., 2013). In addition, unlike cKP infections, approximately half of all hvKP infections occur in young, healthy individuals (Shon et al., 2013; Struve et al., 2015; Paczosa and Mecsas, 2016). The hvKP strain is hypermucoviscous typically because of overproduction of its polysaccharide capsule (Paczosa and Mecsas, 2016). Unlike the typical nosocomial infections of cKP, hvKP can cause serious community-acquired infections in healthy individuals (Patel et al., 2014).

hvKP was reported initially in Taiwan and Southeast Asia in the mid-1980s and 1990s, but more than 30 cases have been reported worldwide (Shon et al., 2013). Although hvKP can cause pneumonias or lung abscesses, it is primarily associated with pyogenic liver abscesses. hvKP is now the most common cause of pyogenic liver abscesses in Asia (Siu et al., 2012; Wang et al., 2013). hvKP is rarely resistant to commonly used antimicrobial agents, except for an intrinsic resistance to ampicillin (Zhang et al., 2016). However, along with the global dissemination of mobile genetic elements conferring antibiotic resistance, antibiotic-resistant hvKP isolates are increasingly being reported (Yao et al., 2015; Zhang et al., 2015b; Zhan et al., 2017). Because carbapenem-resistant hvKP strains may cause severe, untreatable infections in healthy individuals, the emergence of these strains poses a great threat to public health. In this review, we summarize the epidemiology of hvKP isolates and discuss the resistance mechanisms involved.

## Epidemiology of hvKP

The hypermucoviscosity/hypermucoviscous phenotype of hvKP is typically due to the increased production of capsular polysaccharide and the presence of specific virulence genes, such as *rmpA* (Siu et al., 2011; Paczosa and Mecsas, 2016). The hypermucoviscosity phenotype is generally determined by “the string test.” The string test is positive when a viscous string >5 mm in length is formed by stretching bacterial colonies on an agar plate (Fang et al., 2004). Because there are *K. pneumoniae* strains that are hypervirulent, but not hypermucoviscous (Catalán-Nájera et al., 2017), reports about non-hypermucoviscous hvKP types are included in our review (Table 1 and Table S1).

**Table 1:** Epidemiology of antibiotic-resistant hvKP.

**Country**	**Infection type(s)**	**ST(s)**	**Capsule type(s)**	**Virulence loci**	**Resistance mechanism(s)**	**References**
China	VAP	ST11	K47	*ybt, iucABCD*-*iutA*, and *rmpA* (deletion variant of pLVPK)	*bla*_KPC−2_ (*n* = 5)	Gu et al., 2017
	Pneumonia, urinal tract infection	ST11, ST268, ST65, ST692, and ST595	K2 and K20	*rmpA, iucABCD*-*iutA entB*, and *ybtS*	*bla*_KPC−2_ (*n* = 21) *bla*_SHV−11_ (*n* = 20)	Zhan et al., 2017
	Bacteremia, liver abscesses	Unknown	K1 and K2	*rmpA* and *iucABCD*-*iutA*	*bla*_KPC−2_ (*n* = 1) ESBL (*n* = 5)	Wu et al., 2017
	Liver abscess, sepsis, and invasive infections	ST11, ST437, ST23, ST65, and ST86	K1, K2, and K20	*rmpA*	ESBL (*n* = 29)	Zhang et al., 2016
	Diarrhea	ST661	K1	Unknown	*mcr-1* (*n* = 1)	Gu et al., 2016
	Catheter-associated bacteremia	ST11	K1	*magA, rmpA, rmpA2, iro*, and *iucABCD*-*iutA*	*bla*_KPC−2_ (*n* = 1)	Wei et al., 2016
	Pneumonia	ST23	K1, K2, and K20	*rmpA, iro*, and *iucABCD*-*iutA*	ESBL (*n* = 1)	Yan et al., 2016
	Bacteremia, abdominal infection, and septic arthritis	ST23 and ST1797	K1	*magA*, and *rmpA*	*bla*_KPC−2_ (*n* = 5)	Zhang et al., 2015a
	Pneumonia, abdominal infection, septicemia	ST65	K2	*rmpA, iucABCD*-*iutA*, and *entB*	*bla*_KPC−2_ (*n* = 1)	Zhang et al., 2015b
	UTI, pneumonia, septicemia	ST25, ST65, and ST11	K2 and non-typeable	*rmpA, iucABCD*-*iutA*, and *iro*	*bla*_KPC−2_ (*n* = 7)	Yao et al., 2015
	Bacteremia	ST23 and ST1265	K1, K2, K20, and K57	*rmpA*	ESBL (*n* = 2)	Liu et al., 2014
	Liver abscess and other infections	Unknown	K1 and K2	*rmpA*	ESBL (*n* = 5)	Li et al., 2014b
Singapore	Pneumonia, UTI, or other infections	Unknown	non-K1/K2	*rmpA*, and *rmpA2*	ESBL (*n* = 19)	Yu et al., 2017
Taiwan	Invasive syndrome	Unknown	Unknown	*rmpA* and *rmpA2*	ESBL (*n* = 1)	Lee et al., 2010
France	Bacterial infection	ST86	K2	*rmpA, iucABCD*-*iutA, entB*, and *ybtS*	ESBL (*n* = 1)	Surgers et al., 2016
Italy	Liver abscess	ST512	Unknown	Unknown	*bla*_KPC−3_ (*n* = 1)	Arena et al., 2017
Brazil	Various infection sites	ST11	Unknown	Unknown	*bla*_KPC−2_ (*n* = 1)	Andrade et al., 2014

*entB, iron siderophore enterobactin; ESBL, extended-spectrum-β-lactamase; hvKP, hypervirulent Klebsiella pneumoniae; iro, iron siderophore salmochelin; iucABCD-iutA, hydroxamate iron siderophore aerobactin; magA, mucoviscosity-associated gene A; rmpA and rmpA2, regulator of mucoid phenotype A; ST, sequence type; UTI, Urinary tract infection; VAP, ventilator-associated pneumonia; ybtS, iron siderophore yersiniabactin*.

**Figure 1:**
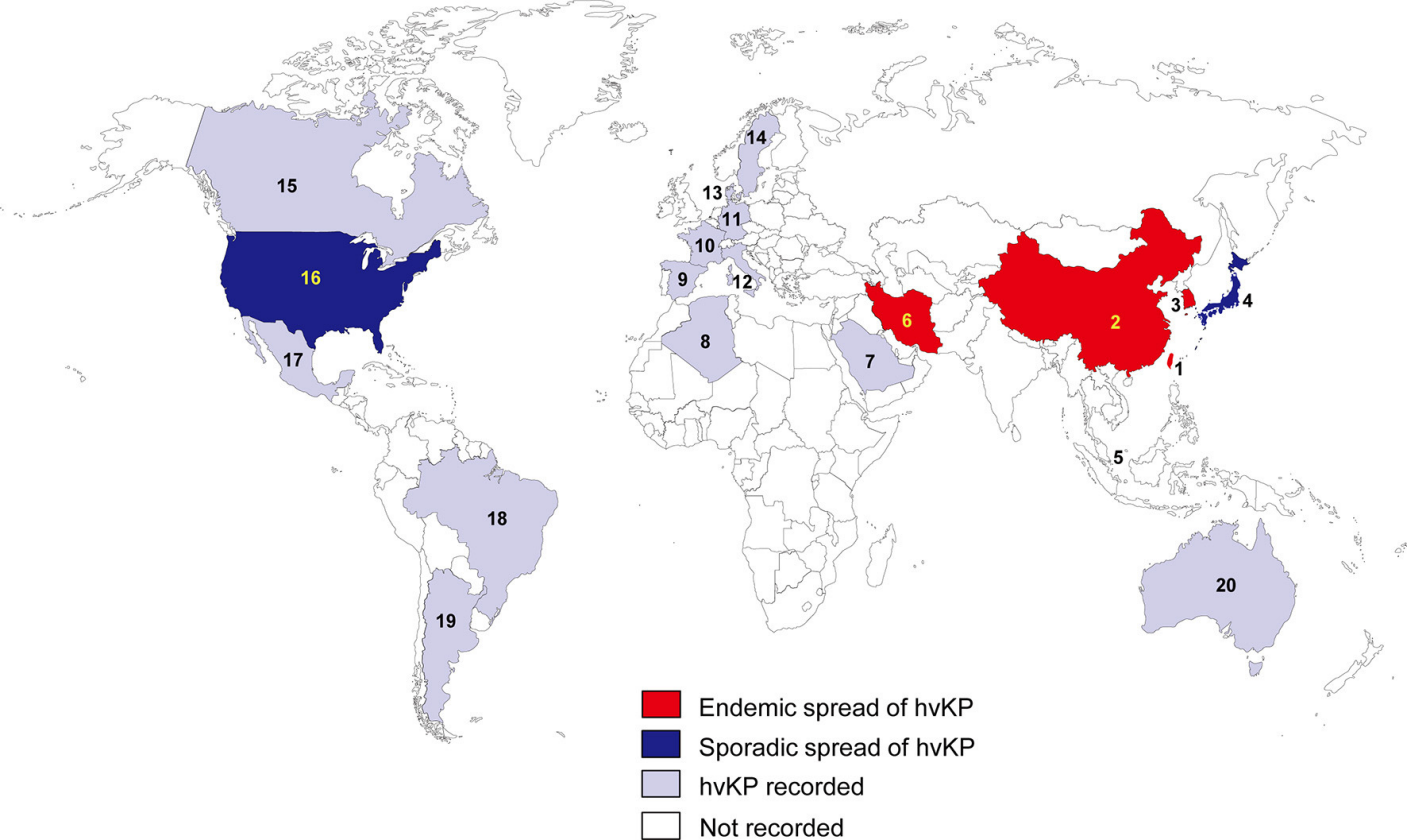
Epidemiological features of hvKP. The endemic spread of hvKP means that multiple outbreaks of hvKP were reported in an indicated region. The sporadic spread of hvKP means that only case studies (no outbreak) were reported in an indicated region. 1, Taiwan; 2, China; 3, South Korea; 4, Japan; 5, Singapore; 6, Iran; 7, Saudi Arabia; 8, Algeria; 9, Spain; 10, France; 11, Germany; 12, Italy; 13, Denmark; 14, Sweden; 15, Canada; 16, United States; 17, Mexico; 18, Brazil; 19, Argentina; 20, Australia.

In the 1980s, case reports from Taiwan described community-acquired liver abscesses caused by hvKP in healthy patients with serious concomitant end-organ manifestations, such as meningitis and endophthalmitis (Liu et al., 1986; Chiu et al., 1988). Since the first report in Taiwan, the sporadic spread of hvKP has been observed in many Asian, European, and American countries (Figure 1). Although several cases of hvKP were reported in Europe and the Americas, the epidemic spread of hvKP occurred primarily in Asian countries, including Taiwan, China, South Korea, and Iran (Table S1). For example, in China, 22.8% (84/369) of *K. pneumoniae* clinical isolates associated with various types of invasive infections were identified as hvKP (Guo et al., 2017). Another report showed that 90.9% of the pathogens causing pyogenic liver abscesses were hvKP (Ye et al., 2016). In South Korea, 42.2% of *K. pneumoniae* strains isolated from patients with bacteremia were of the hypermucoviscosity phenotype (Jung et al., 2013). In Taiwan, 88.8% of *K. pneumoniae* isolates collected from patients with community-acquired extrahepatic abscesses had the hypermucoviscosity phenotype (Ku et al., 2008) and 41.5% of community-acquired *K. pneumoniae* bacteremia were caused by hvKP (Lee et al., 2006). Therefore, *K. pneumoniae* liver abscess is now considered as an endemic disease in Taiwan. The frequency of hvKP-associated diseases in these countries has continued to increase. Between 1996 and 2004, an almost 60% increase in the annual incidence of liver abscesses caused by hvKP were observed in Taiwan (Tsai et al., 2008). In South Korea, the rate of liver abscesses caused by *K. pneumoniae* rose 3.3% in the 1970s to 78.2% in the mid-2000s (Chung et al., 2007). A study in Iran showed that 60.95% of *K. pneumoniae* isolates collected from various patients were hypermucoviscosity-positive (Zamani et al., 2013). Although the reasoning for the hvKP predominance in Asia is unclear (Shon et al., 2013), some reports analyzing the hvKP mechanistic properties have provided clues. A study in 2002 analyzed the phenotypic and genotypic characteristics of *K. pneumoniae* isolates collected worldwide (Ko et al., 2002). Interestingly, a distinctive form of *K. pneumoniae*, often causing liver abscess, was identified almost exclusively from Taiwan (Ko et al., 2002), indicating that the spread of a specific hvKP clone may be the major factor of high predominance in Asia. Another analysis showed that hvKP infections in Western countries most commonly occur in Asians (Lederman and Crum, 2005; Keynan et al., 2007; Nadasy et al., 2007; Pastagia and Arumugam, 2008; Frazee et al., 2009; Gunnarsson et al., 2009; McCabe et al., 2010; Sobirk et al., 2010; Decré et al., 2011; Vila et al., 2011; Pomakova et al., 2012; Shon et al., 2013), suggesting that Asians may be more susceptible to hvKP infections than other ethnic groups. Because Asians in Western countries may have traveled to Asia or been exposed to individuals who recently traveled to Asia, these results do not conclusively establish a genetic risk of Asians for hvKP infections.

In other regions of the world, the prevalence of hvKP is not high (Table S1). Studies from other regions are mostly case reports, but there are several studies analyzing various hvKP isolates. For example, a study in Spain showed that 5.4% of *K. pneumoniae* isolates collected from various infection sites had the hypermucoviscosity phenotype (Cubero et al., 2016). This rate is relatively low, compared with the high frequency (up to 90%) of hvKP in Asia. Similar results were obtained in Canada, Brazil, and Algeria (El Fertas-Aissani et al., 2013; Peirano et al., 2013; Pereira and Vanetti, 2015). In Canada, the hypermucoviscosity phenotype was present in 8.2% of *K. pneumoniae* isolates collected from patients with community-acquired bacteremia (Peirano et al., 2013). In Brazil, the hypermucoviscosity phenotype was observed in 6.7% of clinical *K. pneumoniae* isolates (Pereira and Vanetti, 2015). A study in Algeria showed that 9.2% of *K. pneumoniae* isolates obtained from various patients were hypermucoviscous-positive (El Fertas-Aissani et al., 2013). These results support the theory that the endemic spread of hvKP is mostly in Asian countries.

## Factors associated with hypervirulence of hvKP

A variety of hypervirulence-associated factors are important in hvKP strains, including capsular serotypes, sequence types, a virulence plasmid, a pathogenicity island, and several virulence factors.

### K1 and K2 capsular serotypes

The capsule is an important virulence factor of encapsulated pathogens, including *K. pneumoniae*. Capsular polysaccharides of *K. pneumoniae* are divided into at least 78 serotypes (Pan et al., 2008; Wyres et al., 2016). Notably, many reports have shown that K1 and K2 serotypes are strongly associated with hvKP (Paczosa and Mecsas, 2016; Catalán-Nájera et al., 2017) (Table 1 and Table S1). In China, among the hypermucoviscous *K. pneumoniae* clinical isolates associated with various types of invasive infections, including pyogenic liver abscess, 42.9% were the K2 serotype and 23.8% were the K1 serotype (Guo et al., 2017). Another study in China also showed that 68.75% of the hypermucoviscosity-positive *K. pneumoniae* isolates have the K2 serotype (Zhao et al., 2016). A South Korea study searching *K. pneumoniae* strains isolated from the urine of hospitalized patients showed that K1 and K2 capsular serotypes were more common in hypermucoviscosity-positive strains than in hypermucoviscosity-negative strains (Kim et al., 2017). Most hypermucoviscous *K. pneumoniae* isolates from patients with bacteremia also had the K1 or K2 serotype (78%) (Jung et al., 2013). Many hvKP isolates reported in Europe and America also had the K1 or K2 serotype (Table S1).

Why are the K1 and K2 serotypes so prevalent in hvKP? Two studies tried to resolve this question (Yeh et al., 2007; Lin et al., 2014). Serotype K1 and K2 *K. pneumoniae* isolates demonstrated significantly more virulence in the mouse model than did non-K1/K2 isolates (survival rates of 0 vs. 79.2%, respectively) (Yeh et al., 2007). Typically, K1 and K2 strains are more resistant to phagocytosis and intracellular killing by macrophages and neutrophils than strains with other serotypes, regardless of their hypermucoviscosities (Kabha et al., 1995; Lin et al., 2004; Lee et al., 2014; Paczosa and Mecsas, 2016). Several reasons were suggested for enhanced virulence of the K1 and K2 strains, relative to other strains. First, K1 and K2 strains lack the specific mannose residue repeats that are recognized by the host factors, such as the mannose-binding receptor on macrophage and the lung-secreted surfactant protein A (SP-A) (Kabha et al., 1995; Sahly et al., 2008). Second, K1 and K2 strains have a host-specific monosaccharide sialic acid on their surfaces (Lee et al., 2014), that is known to mimic host cells, thereby allowing evasion of the host immune cells. Third, K1 and K2 strains may induce a smaller release of reactive oxygen species by neutrophils than strains with other serotypes, allowing better survival in human tissues (Sahly et al., 2004; Paczosa and Mecsas, 2016). Fourth, K1 and K2 strains have more diverse O serotypes, compared with strains with other K serotypes (Follador et al., 2016), which may help K1 and K2 strains evade host immune systems. The O serotyping is based on the recognition of distinct variations of O-antigens, the outermost part of the lipopolysaccharide (LPS) (Trautmann et al., 1997).

Although the K1 and K2 serotypes are typically found in hvKP, several strains of cKP also possess these serotypes. At the same time, some hvKP strains have a non-K1 or -K2 serotype (Fang et al., 2007; Shon et al., 2013; Guo et al., 2017). In addition to K1 and K2 capsular serotypes, other capsular serotypes, including K5, K16, K20, K28, K54, K57, K63, and KN1, have also been found in hvKP (Table 1 and Table S1). A study compared the prevalence and importance of virulence factors for *K. pneumoniae* liver abscesses between isolates with capsular K1/K2 serotypes and isolates with non-K1/K2 serotypes (Yu et al., 2008). When injected into mice intraperitoneally, *K. pneumoniae* isolates with the hypermucoviscosity phenotype as well as presence of *rmpA* and *iucABCD*-*iutA* genes exhibited high virulence for mouse lethality, regardless of capsular type (Yu et al., 2008). However, in other reports, K1/K2 cKP strains were not significantly less virulent than K1/K2 hvKP strains (Lin et al., 2004), and serotype K1 and K2 isolates demonstrated significantly more phagocytic resistance and virulence than non-K1/K2 strains, regardless of the presence of the *rmpA* gene (Yeh et al., 2007), suggesting that the presence of the K1/K2 serotypes themselves is responsible for increased virulence. Therefore, these studies indicate that the presence of the K1/K2 serotypes themselves is partially responsible for the hypervirulence of the hvKP strains and the combination with other hypervirulence-associated factors may enhance virulence of *K. pneumoniae*.

### ST23 and CC23 sequence types

Recent studies using a 694-gene core genome multilocus sequence typing (MLST) analysis or whole genome-sequencing revealed that K1 hvKP clonal complex 23 (CC23) isolates form a unique clonal lineage, whereas K2 hvKP isolates are genetically more diverse (Bialek-Davenet et al., 2014; Struve et al., 2015). These results indicate that the clonal lineage CC23 has a specific genetic background conferring hypervirulence and fitness. Another study has also revealed that the sequence type 23 (ST23) clone is strongly associated with the K1 capsular serotype in hvKP strains that were isolated specifically from liver abscesses (Siu et al., 2011). Among 47 serotype K1 isolates, 85.1% belonged to ST23, one isolate belonged to ST163 (a single-locus variant of ST23), and two isolates were ST249 (a three-locus variant of ST23) (Siu et al., 2011). In China, ST23 was found to be the most prevalent ST among 69 hypermucoviscous *K. pneumoniae* isolates and was found only among K1 isolates (Guo et al., 2017). Another study in China showed that 57.8% of *K. pneumoniae* strains from liver abscess patients belonged to ST23, and 96.2% of ST23 *K. pneumoniae* isolates were the K1 serotype (Qu et al., 2015). In South Korea, ST23 strains accounted for 97.3% of hvKP strains with the K1 serotype collected from patients with community-acquired liver abscesses (Chung et al., 2008). Similar results were found in many Western countries (Table S1). Reasons for the association of the K1 serotype with ST23 are unclear. Meanwhile, hvKP strains of the K2 serotype are genetically diverse (Shon et al., 2013). A recent study analyzing serotype K2 isolates from three different regions in Asia showed that eight different sequence types were found among serotype K2 isolates (ST65, ST66, ST86, ST373, ST374, ST375, ST380, and ST434) (Lin et al., 2014). There were two major groups, ST65-like (42%) and ST86-like (46%) (Lin et al., 2014). Notably, ST23 hvKP clones with the K1 serotype were associated with pyogenic liver abscess, whereas ST65 hvKP clones with the K2 serotype were correlated with various invasive infections (Guo et al., 2017).

Whole genome-sequencing of hvKP isolates from different geographic origins revealed the epidemiology of the hvKP CC23 expansion. Only minimal geographical grouping of the isolates was observed in the CC23 phylogeny, suggesting that CC23 may spread worldwide through multiple international transmission events rather than by local expansions (Struve et al., 2015). Indeed, infections of hvKP linked to recent travels to Asia were reported in several cases from Western countries (Fang et al., 2005; Gunnarsson et al., 2009; Rivero et al., 2010). However, because there were many cases not linked to travel or close contact with individuals from epidemic regions (Nadasy et al., 2007; Doud et al., 2009; Frazee et al., 2009; Pinsky et al., 2009; Decré et al., 2011; Vila et al., 2011; Merlet et al., 2012; Pomakova et al., 2012; Bilal et al., 2014; Coutinho et al., 2014; Gundestrup et al., 2014; Holmås et al., 2014; Melot et al., 2016; Surgers et al., 2016), an independent local establishment of the CC23 lineage outside of Asia is also possible.

### The virulence plasmid pLVPK and KPHP1208 pathogenicity Island

Notably, a large virulence plasmid (pLVPK, a pK2044-like plasmid) was detected in all whole genome-sequenced hvKP clonal lineages (Struve et al., 2015). This plasmid encodes aerobactin, salmochelin, and RmpA, which were found to be restricted to hvKP isolates (Struve et al., 2015). A CC23 strain lacking pLVPK was found to have significantly reduced virulence (Lin et al., 2011, 2012), indicating an important role of this plasmid in the hypervirulence of *K. pneumoniae*.

Additionally, a genomic comparison revealed a distinct variant of a pathogenicity island (KPHP1208) specifically associated with CC23 (Struve et al., 2015). Most CC23 isolates contain genes encoding yersiniabactin, colibactin, and microcin E492, in the KPHP1208 pathogenicity island (Struve et al., 2015). Colibactin is a polyketide-peptide genotoxin that causes DNA damage in eukaryotic cells (Lai et al., 2014). A recent study using a meningitis mouse model showed that colibactin was necessary for the meningeal tropism of K1 CC23 *K*. *pneumoniae* (Lu et al., 2017), indicating the importance of the KPHP1208 genomic island in the hypervirulence of *K. pneumoniae*.

### RmpA and MagA

RmpA (*r*egulator of the mucoid phenotype A gene) activates capsule production, resulting in the hypermucoviscosity phenotype and increase in virulence (Cheng et al., 2010). Three *rmpA* genes were reported in hvKP strains: two large plasmid-carried genes (p-*rmpA* and p-*rmpA2*) located in a virulence plasmid (pLVPK) and one chromosomal gene (c-*rmpA*) (Wacharotayankun et al., 1993; Hsu et al., 2011). In *K. pneumoniae* CG43, both p-*rmpA* and p-*rmpA2* contributed to the enhancement of capsular production (Lai et al., 2003; Cheng et al., 2010), whereas in NTUH-K2044 (ST23, K1 serotype), the first genome-sequenced hvKP, only p-*rmpA*, but not p-*rmpA2* or c-*rmpA*, contributed to the increased expression of capsule genes (Hsu et al., 2011). Therefore, additional studies in other strains are needed to clarify the role of each RmpA in hypervirulence.

Many results have revealed a strong association between RmpA and hypervirulence (Table 1 and Table S1). Whole-genome sequencing of 30 hvKP isolates from different geographic origins revealed that all hvKP were *rmpA* positive (Struve et al., 2015). Several studies from China also showed that 92–100% of hvKP isolates were positive for *rmpA* (Liu et al., 2014; Sun et al., 2016; Wu et al., 2017). Regardless of the investigated infection site, most hvKP isolates appear to have the *rmpA* gene (Ku et al., 2008; Yu et al., 2008; Lee et al., 2010; Decré et al., 2011; Qu et al., 2015; Guo et al., 2016; Zhang et al., 2016; Wu et al., 2017; Zhan et al., 2017). Despite the strong association between hvKP and RmpA, some *rmpA*-positive isolates did not exhibit the hypermucoviscosity phenotype (Yu et al., 2006). However, recent studies have shown that the lack of hypermucoviscosity and low virulence in some *rmpA*-positive *K. pneumoniae* strains were caused by a concurrent mutation of *rmpA* and *rmpA2* genes in the absence of c-*rmpA* (Yu et al., 2015a,b).

The *magA* gene was discovered in 2004 as a gene required for the hypercapsular phenotype from *K. pneumoniae* strains isolated from invasive liver abscesses (Fang et al., 2004). Subsequent works using bioinformatic analysis and genetic experiments determined that *magA* is the serotype K1 allele of the polymerase gene *wzy* in the *cps* gene cluster (Fang et al., 2007; Yeh et al., 2010). Consequentially, *magA* was renamed *wzy*_K1 (Fang et al., 2010). A study in Taiwan showed that 83% of *K. pneumoniae* strains from patients with pyogenic liver abscess were *magA* positive, whereas only 3% of strains isolated from patients with other invasive infections were *magA* positive (Chuang et al., 2006). Additionally, all 36 *magA*-positive strains were identified as serotype K1, whereas the 38 *magA*-negative strains were not serotype K1 (Chuang et al., 2006). Another study also reported the association of *magA* with the capsule serotype K1 (Yeh et al., 2006). A recent study showed that *magA* was found more prevalently in hvKP strains with the K1 serotype than in non-K1 strains (Guo et al., 2017). These results suggest that the *magA* gene is responsible for the *K. pneumoniae* capsular serotype K1.

### Aerobactin

Iron is a metal that is essential for bacterial growth and plays a crucial role in the progression of infection, including *K. pneumoniae* infection (Russo et al., 2014). *K. pneumoniae* can secrete various siderophores (aerobactin, salmochelin, enterobactin, and yersiniabactin) that acquire iron in iron-depleted environments, such as in a human host (Hsieh et al., 2008). The hvKP strains have a 6- to 10-fold increased siderophore activity compared with cKP strains (Russo et al., 2011, 2014). Enterobactin is a siderophore that is produced by more than 90% of examined enterobacterial isolates (Raymond et al., 2003), whereas genes encoding aerobactin and salmochelin are located on a large virulence plasmid (pLVPK) that is not present in most cKP strains (Nassif and Sansonetti, 1986; Struve et al., 2015) and the yersiniabactin locus located within the pathogenicity island of *Yersinia* species is believed to be acquired via horizontal gene transfer (Bach et al., 2000). Therefore, aerobactin and salmochelin were rarely expressed in cKP strains, but it was present in most hvKP isolates (Paczosa and Mecsas, 2016). Interestingly, although hvKP secretes four different siderophores, a study showed that aerobactin accounts for >90% of the siderophore activity (Russo et al., 2014). Furthermore, a recent study revealed that aerobactin, but not salmochelin, enterobactin, or yersiniabactin, plays a crucial role in the growth and survival of hvKP in human ascites fluid or serum and in the *in vivo* mouse infection models (Russo et al., 2015), suggesting that aerobactin is a crucial virulence factor of hvKP. Aerobactin is encoded by the *iucABCD* operon and its cognate receptor is encoded by the *iutA* gene (Russo et al., 2014).

Many reports showed that an *iucABCD*-*iutA* gene was more common in hvKP strains than in cKP strains (Table 1 and Table S1). In China, many studies have reported the high prevalence (69–96%) of aerobactin in hvKP isolates (Guo et al., 2016, 2017; Sun et al., 2016; Yan et al., 2016; Ye et al., 2016; Zhang et al., 2016; Zhao et al., 2016; Wu et al., 2017; Zhan et al., 2017). A study in Taiwan also showed that the prevalence of aerobactin was 100% in capsular K1 and K2 isolates and 86% in non-K1/K2 isolates. Furthermore, in this study, all *K. pneumoniae* isolates with *rmpA* and *iucABCD*-*iutA* genes in the presence of the hypermucoviscosity phenotype exhibited high virulence for mouse lethality, regardless of capsular type (Yu et al., 2008). When serotype K1 *K. pneumoniae* isolates from Hong Kong, Singapore, and Taiwan were analyzed, all hypervirulent isolates with a 50% lethal dose of <10^2^ colony-forming units were identified as ST23 and carried both virulence-associated genes, *rmpA* and *iucABCD*-*iutA* (Siu et al., 2011). These results imply the importance of *iucABCD*-*iutA* and *rmpA* genes for hypervirulence. Notably, the expression of *rmpA* is regulated by the availability of iron and is negatively controlled by Fur, the iron-responsive global regulator (Cheng et al., 2010), implying that the availability of iron regulates the expression of *rmpA*, which could affect virulence of *K. pneumoniae*. Therefore, additional studies are needed to clarify the relationship between aerobactin and RmpA in hypervirulence.

## Antibiotic resistance of hvKP

The prevalence of antibiotic resistance in hvKP isolates is rare compared with the high prevalence of antibiotic-resistant cKP isolates (Lee et al., 2016; Paczosa and Mecsas, 2016). The reason for the low prevalence of antibiotic resistance in hvKP strains remains unclear; however, reports of antibiotic-resistant hvKP isolates are increasingly being reported worldwide, mostly in countries with an epidemic dissemination of hvKP (Table 1). Therefore, we will summarize and discuss the epidemiology and resistance mechanisms of antibiotic-resistant hvKP strains.

### Antibiotic-resistant hvKP in Asia

Except for an intrinsic resistance to ampicillin, most hvKP strains are rarely resistant to antibiotics and are generally susceptible to commonly used antimicrobial drugs (Paczosa and Mecsas, 2016; Zhang et al., 2016). However, with the global dissemination of mobile genetic elements harboring various antibiotic resistance genes, including the *K. pneumoniae* carbapenemase (KPC), NDM, and oxacillinases-48 (OXA-48) types of carbapenemases (Lee et al., 2016), antibiotic-resistant hvKP isolates have begun to emerge in the past few years (Zhang et al., 2015b). In 2010, a study from Taiwan analyzed various features of isolates with and without the hypermucoviscosity phenotype (Lee et al., 2010). The prevalence rates of extended-spectrum β-lactamase (ESBL) were significantly higher in *K. pneumoniae* isolates without the hypermucoviscosity phenotype (34%) than in isolates with the hypermucoviscosity phenotype (4%), and only one ESBL-producing strain among the 24 isolates with the hypermucoviscosity phenotype was detected (Lee et al., 2010). Several ESBL-producing hvKP strains causing bloodstream infections were found in China (Liu et al., 2014; Yan et al., 2016). Another report in China showed that 17% of hvKP strains expressed ESBL and several hvKP isolates showed resistance to all the tested antimicrobials, except carbapenems and amikacin (Li et al., 2014b). Furthermore, antimicrobial resistance in hvKP strains increased over time (Li et al., 2014b). Like the report in Taiwan, the resistance to antibiotics in China was found to be significantly greater in cKP strains than in hvKP strains (Li et al., 2014b). Similarly, a report in 2016 showed that 12.6% of hvKP isolates from several invasive infections produced ESBLs, and most of them carried *bla*_CTX−M_ genes (Zhang et al., 2016).

Recently, in China, the prevalence of hvKP in invasive infections was studied in several reports. In a study in 2016, 90.9% of the pathogens causing pyogenic liver abscess were hvKP (Ye et al., 2016). A study analyzing 369 *K. pneumoniae* isolates associated with various invasive infections revealed that 22.8% (84/369) of the isolates were identified as hvKP (Guo et al., 2017). With a national prevalence of hvKP, several studies in China have revealed the emergence of hvKP that have acquired extensive antibiotic resistances, including carbapenem resistance. In 2015, among 33 patients with carbapenem-resistant *K. pneumoniae*, carbapenem-resistant hvKP isolates were found in four patients (Yao et al., 2015). From these four patients, seven carbapenem-resistant hvKP isolates were identified and the *bla*_KPC−2_ gene was detected in six of the seven hvKP isolates (Yao et al., 2015). Notably, carbapenem-resistant hvKP strains belonged to three sequence types, [ST65 (five isolates), ST25 (one isolate), and ST11 (one isolate)], and two capsular serotypes, [K2 (six isolates) and non-typeable (one isolate)] (Yao et al., 2015). A retrospective study analyzing 28 cases of carbapenem-resistant *K. pneumoniae* infections from nine cities in China showed a similar result. Among the 28 carbapenem-resistant *K. pneumoniae* isolates, five carbapenem-resistant hvKP isolates were detected and three isolates had the *bla*_KPC−2_ gene (Zhang et al., 2015b). Interestingly, the sequence types of carbapenem-resistant hvKP strains were ST11 (three isolates), ST65 (one isolate), and ST1700 (one isolate). The serotypes were K2 (one isolate) and non-typeable (four isolates) (Zhang et al., 2015b). A recent study analyzed a total of 1,838 *K. pneumoniae* isolates collected from various patient specimens of patients between 2013 and 2015. Results were similar, identifying 21 carbapenem-resistant hvKP isolates, with all 21 isolates positive for *bla*_KPC−2_ (Zhan et al., 2017). Additionally, all the carbapenem-resistant hvKP isolates except one harbored *bla*_SHV−11_. Notably, the carbapenem-resistant hvKP strains belonged to five sequence types, [ST11 (16 isolates), ST65 (one isolate), ST268 (two isolates), ST595 (one isolate), and ST692 (one isolate)], and three capsular serotypes, [K20 (six isolates), K2 (one isolate), and non-typeable (14 isolates)] (Zhan et al., 2017). In a 2016 case report in China, the carbapenem-resistant ST11 hvKP strain was detected (Wei et al., 2016). This strain also harbored *bla*_KPC−2_. In conclusion, the prevalence of hvKP among carbapenem-resistant *K. pneumoniae* isolates in China have significantly been high, ranging from 7.4 to 15% (Zhang et al., 2015a; Zhan et al., 2017).

Carbapenem-resistant ST23 hvKP strains with the K1 serotype have not often been detected in China. Most of the identified carbapenem-resistant hvKP strains have the *bla*_KPC−2_ gene. However, a study in 2016 revealed the detection of carbapenem-resistant ST23 hvKP strains with the K1 serotype. A retrospective study in 2016 was conducted on 148 meropenem-resistant *K. pneumoniae* strains collected from two hospitals in Zhejiang Province in 2013, and among these isolates, seven K1, one K2, and five non-K1/K2 hvKP strains were identified by the loop string test (Zhang et al., 2015a). Among them, seven carbapenem-resistant hvKP strains with the K1 serotype belonged to only two sequence types, ST23 (four isolate) and ST1797 (three isolates); five strains harbored *bla*_KPC−2_ (Zhang et al., 2015a). In this study, the *bla*_KPC−2_ gene also was detected in most carbapenem-resistant hvKP strains (Zhang et al., 2015a). In China, the KPC types of carbapenemases are more prevalent than other global carbapenemases, such as NDM and OXA-48 types (Lee et al., 2016). KPC-2 was the most frequently identified carbapenemase in *K. pneumoniae* in many countries, including China (Gupta et al., 2011; Munoz-Price et al., 2013; Li et al., 2014a; Lee et al., 2016), and ST11, which is rarely found in hvKP strains (Liu et al., 2014; Zhang et al., 2016; Table 1), is the prevalent clone associated with the spread of KPC-producing *K. pneumoniae* in Asia (particularly in China and Taiwan) (Qi et al., 2011; Lee et al., 2016). Therefore, these epidemiological features of carbapenemase-producing *K. pneumoniae* in China imply the emergence of carbapenem-resistant hvKP strains by interaction between cKP and hvKP strains.

In China, the predominant clone of carbapenem-resistant *K. pneumoniae* is KPC-2-producing ST11 (Qi et al., 2011; Lee et al., 2016) and ST23 is one of the dominant clones of hvKP (Table 1). Among 36 carbapenem-resistant hvKPs identified in China at the present time (Yao et al., 2015; Zhang et al., 2015a,b; Wei et al., 2016; Wu et al., 2017; Zhan et al., 2017), the rates of KPC-2-producing ST11 and KPC-2-producing ST23 clones are 50% (18/36) and 8.3% (3/36), respectively. Notably, KPC-2 is found in 94.4% (34/36) of carbapenem-resistant hvKP isolates. These results imply that the exchange of mobile genetic elements between KPC-2-producing ST11 cKP and ST23 hvKP has occurred. Additionally, because the rate (50%) of KPC-2-producing ST11 hvKP isolates is far higher than that of KPC-2-producing ST23 hvKP (8.3%), the transmission of hypervirulence-related mobile genetic elements, such as the pLVPK plasmid, from ST23 hvKP strains to KPC-2-producing ST11 cKP strains seems to be a prevalent event. Of course, this assumption should be verified by many additional carbapenem-resistant clinical isolates of hvKP, because the number of currently identified isolates is too small (36 isolates). A study in 2014 showed the *in vitro* transmission and retainment of a plasmid carrying *bla*_KPC−2_ from a parental non-serotype ST258 *K. pneumoniae* to a recipient invasive virulent K2 ST65 *K. pneumoniae* strain (Siu et al., 2014). However, the *in vitro* transmission of plasmids with hypervirulence-related genes, such as pLVPK, from the parental serotype K1 ST23 *K. pneumoniae* to the recipient carbapenem-resistant ST11 *K. pneumoniae* strain has not been reported yet. Notably, a recent study in China showed that five carbapenem-resistant ST11 hvKP strains had the pLVPK-like plasmid (Gu et al., 2017). Genomic analyses showed that the emergence of these ST11 carbapenem-resistant hvKP strains was due to the acquisition of the pLVPK-like plasmid by ST11 carbapenem-resistant cKP strains (Gu et al., 2017). Therefore, additional studies are needed to verify the transmission of mobile genetic elements between cKP and hvKP strains.

94.4% of carbapenemases found in carbapenem-resistant hvKP isolates in China are KPC-2. KPC-2 was the most prevalent carbapenemase in *K. pneumoniae* in China (Li et al., 2014a; Lee et al., 2016). The global epidemiology of carbapenem-resistant *K. pneumoniae* showed that the most prevalent carbapenemase of each country varies (Lee et al., 2016). For example, the most prevalent carbapenemases of Pakistan and Turkey is NDM and OXA-48, respectively (Lee et al., 2016). If the most prevalent carbapenemase of carbapenem-resistant hvKPs in Pakistan and Turkey are identified as NDM and OXA-48, respectively, then these results suggest the necessity of investigating the possibility for the direct transmission of mobile genetic elements between cKP and hvKP. In any case, results from China caution researchers and clinicians of the potential for the emergence and global dissemination of hvKP strains with acquired carbapenem resistance.

Colistin (polymyxin E) is regarded as a key component for the combination antimicrobial therapy used for the treatment of severe carbapenem-resistant *K. pneumoniae* infections (Lee et al., 2016). However, many recent studies have shown that some strains acquire colistin resistance through LPS modification (Cannatelli et al., 2013, 2014), and the frequency of these colistin-resistant isolates is gradually increasing (Giani et al., 2015; Lee et al., 2016). Particularly, the emergence of a plasmid carrying the *mcr-1* gene, which mediates colistin resistance by modifying LPS, was reported in China (Liu et al., 2016). A recent study in China reported the detection of an hvKP isolate harboring the *mcr-1* gene (Gu et al., 2016). This isolate was identified from a stool specimen of an infant patient with diarrhea in China and was identified as the ST661 clone with the K1 serotype (Gu et al., 2016). This particular case indicates that hvKP can acquire various types of antibiotic resistances.

A recent study showed that the acquisition of colistin resistance of ST23 hvKP strains was accompanied by reduced capsule production, impaired virulence, and a significant fitness cost (Choi and Ko, 2015). Similar results regarding the effects of colistin resistance on virulence and fitness costs have been observed in other Gram-negative pathogens, including *Acinetobacter baumannii* and *Salmonella enterica* (Sun et al., 2009; López-Rojas et al., 2011; Hraiech et al., 2013; Beceiro et al., 2014). Although the effect of antibiotic resistance on virulence and fitness costs in hvKP strains has not been assessed for other antibiotics, including carbapenems, high fitness burdens in antibiotic-resistant hvKP strains may explain partially the low prevalence and restricted spread of antibiotic-resistant hvKP strains.

### Antibiotic-resistant hvKP in Europe and America

A few cases of antibiotic-resistant hvKP were reported in Europe and America (Table 1). In France, an ESBL (*bla*_CTX−M−3_)-producing hvKP strain was identified in a patient born in Algeria, who alternately resided in France and Algeria for several years without travel to any other countries (Surgers et al., 2016). The strain was the ST86 clone with the K2 serotype. In Brazil in 2014, a polymyxin B-resistant and KPC-2-producing hvKP was detected (Andrade et al., 2014); the strain was the ST11 clone. KPC-2 is the most prevalent carbapenemase in Brazil (Lee et al., 2016), and the correlation is consistent with cases in China. Recently, a study in Italy showed the detection of a KPC-3-producing hvKP isolate from a 52 year old Caucasian patient with liver abscess (Arena et al., 2017). The sequence type for this strain was ST512 (a single-locus variant of ST258). KPC-type enzymes were the most common carbapenemase in Italy (89.5% of carbapenemase producers), followed by VIM-1 (9.2%) and OXA-48 (1.3%) (Giani et al., 2013; Lee et al., 2016). These results imply the possibility for the direct transmission of mobile genetic elements between cKP and hvKP strains.

### Lack of treatment options for carbapenem-resistant hvKP

Although most hvKP strains are susceptible to antimicrobial drugs, except for ampicillin (Shon et al., 2013), and a fair number of patients with hvKP infection are young with no other illnesses, mortality rates of hvKP infections are high, ranging from 3 to 42% (Han, 1995; Wang et al., 1998; Fang et al., 2000, 2007; Ko et al., 2002; Ku et al., 2008; Decré et al., 2011; Shon et al., 2013). Cases of community-acquired pneumonia with bacteremia and necrotizing fasciitis have presented high mortality rates of 55 and 47%, respectively (Lin et al., 2010; Cheng et al., 2012). Furthermore, survivors of hvKP infections have often suffered catastrophic morbidity such as loss of vision or neurologic sequelae (Liu et al., 1986; Cheng et al., 1991, 2012; Han, 1995; Fang et al., 2007; Shon et al., 2013).

Management of hvKP infections was made more difficult by the emergence of carbapenem-resistant hvKP strains. In cases of 34 patients with carbapenem-resistant hvKPs (Andrade et al., 2014; Yao et al., 2015; Zhang et al., 2015a; Arena et al., 2017; Zhan et al., 2017), 10 patients died and all treatment of 11 patients ceased because of lack of effective antibiotics. Difficulties managing carbapenem-resistant hvKPs could make this strain the next worldwide “superbug” in waiting.

## Conclusions

This review summarizes the epidemiology and antibiotic resistance mechanisms of hvKPs. Invasive hvKPs infections have several dangerous features that are uncommon for cKP. First, approximately half of the patients with hvKP infections are young or do not have co-morbidities. Second, most hvKP infections are community-acquired infections that are unusual for cKP, which are generally nosocomial infections in patients with immunosuppression. Third, hvKP infections are associated with a mortality rate, ranging from 3 to 42%. Fourth, hvKP infections are often complicated by devastating disseminated infections, including endophthalmitis and meningitis, which are uncommon for enteric Gram-negative bacilli, including *K. pneumoniae* in the absence of host susceptibility factors. These pathogenic features of hvKP may be primarily involved in its hypervirulence, but the exact mechanisms remain unclear. Other features of hvKP are its epidemic spread in Asia and the low prevalence of antibiotic resistance. Although hvKP infections have increasingly been reported in Western countries, most cases of hvKP infections were reported along the Asian Pacific Rim, including Taiwan, China, South Korea, Singapore, and Japan (Figure 1). The reasons for the prevalence of hvKP infections in Asia remain unclear. Despite the global prevalence of antibiotic-resistant cKP strains, the rate of antibiotic resistance in hvKP strains is relatively low and the exact reasons for this also remain unclear.

The exact definition of hvKP is a controversial issue. Although the string test is generally accepted as a method for determining hvKP, many cases of string test-positive *K. pneumoniae* isolates do not exhibit hypervirulence. Several virulence factors, such as the K1/K2 serotypes, RmpA, and aerobactin, seem to be associated strongly with the hypervirulence of hvKP. Therefore, the exact phenotypic and genotypic definitions of hvKP need to be established firmly. The predominant hvKP clone is ST23, and most ST23 clones have the K1 serotype. The reason for this also remains unclear. It is interesting that the predominant clone of carbapenem-resistant cKP, ST258, is not the predominant clone of hvKP. Additional studies analyzing epidemiological and pathogenic characteristics of each clone are required to understand the exact relationship between the specific clones and specific phenotypes of *K. pneumoniae*, such as hypervirulence or antibiotic resistance.

The threat of hvKP acquiring carbapenem resistance is becoming a reality in certain countries, such as China, where both carbapenem-resistant cKP and carbapenem-susceptible hvKP strains are prevalent. The prevalence of hvKP among carbapenem-resistant *K. pneumoniae* isolates in China is high, ranging from 7.4% to 15%. The epidemiology of carbapenem-resistant hvKP in China implies the possibility of the transmission of mobile genetic elements between hvKP and cKP strains. Until now, ST11 and ST23 have been identified as the major clones of carbapenem-resistant hvKP strains in China. In China, ST11 and ST23 are the major clones of cKP and hvKP strains, respectively. Additionally, KPC-2 is found in 94.4% of carbapenem-resistant hvKP isolates in China. KPC-2 is the most prevalent carbapenemase in China. These results also imply the transfer of mobile genetic elements between hvKP and cKP strains. A recent study using genomic analyses showed that ST11 carbapenem-resistant cKP strains could become carbapenem-resistant hvKP strains through the acquisition of the pLVPK-like plasmid. Therefore, an immediate response to the global emergence of hvKP with resistance determinants, especially against carbapenems, is required.

In conclusion, great knowledge gaps exist for hvKP, despite a great deal of clinical, physiological, and pathogenic studies of hvKP over the past 30-years. The definition of hvKP remains unclear. Hypervirulence-associated determinants of hvKP require further study. These hypervirulence-associated factors may become a good target for the development of novel therapeutic agents. Epidemiological analysis of recent hvKP clinical isolates in China indicates a potential for the global dissemination of hvKP strains with extensive antibiotic resistances in the near future. The development of effective diagnostic tools for identification of hvKP is required for the effective control of hvKP in the clinical field. The development of novel antimicrobial agents also is absolutely required.

## Author contributions

C-RL, JL, KP, and SL contributed to the conception and the design of the review and C-RL, JL, KP, JJ, YK, C-JC, BJ, and SL researched and wrote the review.

### Conflict of interest statement

The authors declare that the research was conducted in the absence of any commercial or financial relationships that could be construed as a potential conflict of interest.
