# Supplementary material for: Antimicrobial Resistance of Hypervirulent Klebsiella pneumoniae: Epidemiology, Hypervirulence-Associated Determinants, and Resistance Mechanisms
Source: Front Cell Infect Microbiol. 2017 Nov 21;7:483. doi: 10.3389/fcimb.2017.00483 (PMC5702448; doi:10.3389/fcimb.2017.00483)
Supplement: Supplementary file 1 [file Table1.DOCX]

**Table S1 | Epidemiology of antibiotic-susceptible hvKP**

| **Country** | **Type of infection** | **Sequence type** | **Capsule type/ Virulence loci** | **Frequency of hvKP** | **Reference** |
| --- | --- | --- | --- | --- | --- |
| China | Liver abscesses, various types of invasive infections | ST23 was found to be the most prevalent ST among 69 hvKP isolates and was only found among K1 isolates. | K2 (42.9%) was the most common capsular serotype among hvKP isolates, followed by K1 (23.8%); The positive rates of *iutA*, *iucABCD*-*iutA*, *iroN*, and *rmpA* among hvKP isolates were significantly higher than those among cKP isolates; There was a correlation between *magA* and *ybtS* and K1 isolates. | 22.8% of *K. pneumoniae* clinical isolates associated with various types of invasive infections were identified as hvKP. | (Guo et al., 2017) |
|  | Invasive community-acquired pyogenic liver abscess | ST23 | K1 and K2; The report identified 30 genes that were highly associated with liver abscess *K. pneumoniae*, including *iroBCD*, *iucABCD*, *rmpA*/*A2,* and 21 new genes. | 90.9% of the pathogens causing pyogenic liver abscess were hvKP. | (Ye et al., 2016) |
|  | Clinical *K. pneumoniae* isolates | ST65, ST23, ST86, ST412, ST375 | 68.75% of the hvKP strains had K2 serotype and carried the *rmpA* and *iucA* genes. | 24.62% of 65 total clinical isolates were hypermucoviscosity-positive. | (Zhao et al., 2016) |
|  | Liver abscess |  | 92.1% and 84.2% of *K. pneumoniae* strains that cause liver abscess were *rmpA* and *iucABCD*-*iutA* positive, respectively. | 81.6% of *K. pneumoniae* strains that cause liver abscesses were string test positive. | (Sun et al., 2016) |
|  | Ventilator-associated pneumonia | Unknown | The prevalence of *rmpA* and *iucABCD*-*iutA* genes were 85.7 % and 85.7 %, respectively, and serotypes K1 and K2 accounted for 14.3 % and 28.6 % of the hvKP strains, respectively. | Hypermucoviscosity was found in 20 % isolates of *K. pneumoniae*. | (Guo et al., 2016) |
|  | Pneumonia, septicemia | ST86 | K2; *rmpA*, *iucABCD*-*iutA* | Case report | (Zhang et al., 2015) |
|  | Pyogenic liver abscess | 57.8% of *K. pneumoniae* strains belonged to ST23; 96.2% of ST23 *K. pneumoniae* isolates were *magA*-positive and K1 serotype. | 88.9% of *K. pneumoniae* strains belonged to K1/K2 serotype. All *K. pneumoniae* strains were *rmpA* positive, and 68.9% of these strains were *magA* positive. | 28.9% of *K. pneumoniae* isolates exhibited hypermucoviscous phenotype. | (Qu et al., 2015) |
|  | Pyogenic liver abscess | ST23, ST65 | K1, K2; *rmpA*, *iucABCD*-*iutA* | 70.6% of *K. pneumoniae* isolates exhibited hypermucoviscosity phenotype. | (Luo et al., 2014) |
| Japan | Meningitis | ST29 | K54; *rmpA*, *iro*, *iucA* | Case report | (Iwasaki et al., 2017) |
|  | Septic hip arthritis | ST23 | K1; *magA* | Case report | (Kishibe et al., 2016) |
|  | Liver abscess syndrome, lung abscess | Unknown | *magA*, *rmpA* | Case report | (Namikawa et al., 2016) |
|  | Liver abscess | Unknown | K2; *rmpA* | Case report | (Seo et al., 2016) |
|  | Bloodstream infection | Unknown | RmpA gene was associated with the hypermucoviscosity phenotype. | The hypermucoviscosity phenotype was significantly associated with septic shock at the onset of infection. | (Togawa et al., 2015) |
|  | Septic arthritis |  | *rmpA* |  | (Suzuki et al., 2013) |
|  | Necrotizing fasciitis, psoas muscle abscess | Unknown | Unknown | Case report | (Mita et al., 2012) |
|  | Liver abscess | Unknown | *rmpA* | Case report | (Nakamoto et al., 2011) |
| Iran | *K. pneumoniae* isolates collected from patients | Unknown | Unknown | 60.95% of *K. pneumoniae* isolates showed a hypermucoviscosity positive phenotype. | (Zamani et al., 2013) |
| Korea | *K. pneumoniae* isolated from urine | Unknown | The K1 and K2 capsular serotypes were more common in hvKP strains than in cKP strains. The *iucABCD*-*iutA*, *rmpA*, and *rmpA2* genes were more prevalent in hvKP strains than in cKP strains. | Of the 81 *K. pneumoniae* isolates, 12.3% produced a positive string test. | (Kim et al., 2017) |
|  | Ventriculitis | Unknown | K1; *rmpA* | Case report | (Hyun et al., 2014) |
|  | Bacteremia | ST21, ST23, and ST11 | 78% pf hvKP isolates were serotype K1 or K2. The genes *rmpA*, *rmpA2*, and *iucABCD*-*iutA* were significantly more frequent in hvKP than cKP isolates. | 42.4% of *K. pneumoniae* isolates showed the hypermucoviscous phenotype. The hypermucoviscous phenotype was more associated with community-acquired infection. | (Jung et al., 2013) |
| Saudi Arabia | Community-acquired invasive liver abscess with bacteremia and endophthalmitis | Unknown | K1; *rmpA* | Case report | (Enani and El-Khizzi, 2012) |
| Singapore | Liver abscess syndrome | ST23 | K1, K2, K5, K16, K28, K57 and K63; K1 isolates carried higher frequencies of virulence-associated genes, including *rmpA*, *iuc*, *iro*, and *ybtS*, than non-K1 isolates. | Unknown | (Lee et al., 2016) |
| Taiwan | Clinical *K. pneumoniae* isolates | Unknown | Carriage of *rmpA*, *iutC*, and *ybtA* was significantly higher in the *pks*-positive isolates than the *pks*-negative isolates (95.5% vs. 13.2%). | Unknown | (Chen et al., 2017) |
|  | Isolates  recovered from blood and urine cultures | Unknown | *rmpA*, *rmpA2* | Unknown | (Yu et al., 2015a) |
|  | Bloodstream isolates | ST23, ST65, ST86, ST373, ST375 | K1, K2, K5, K57 | Unknown | (Yan et al., 2015) |
|  | Various infection sites | Unknown | *rmpA*, *rmpA2* | The prevalence rates of hypermucoviscosity phenotype as well as *rmpA* and *rmpA2* genes were significantly higher in non-ESBL group than in the ESBL group. | (Yu et al., 2015b) |
|  | Mycotic aneurysm | ST29 | K54; *rmpA*, *rmpA2* | Case report | (Chuang et al., 2013) |
|  | Bacteremic community-acquired pneumonia | Unknown | K1, K2 | All K1/K2 isolates and 20 out of 23 non-K1/K2 isolates were positive for the hypermucoviscosity phenotype, *iucABCD*-*iutA*, and *rmpA* genes. | (Lin et al., 2010b) |
|  | 54 *K. pneumoniae* isolates from patients with community-acquired urinary tract infections | Unknown | Unknown | 27.8% of *K. pneumoniae* isolates had the hypermucoviscosity phenotype. 29.6% of *K. pneumoniae* isolates had the *rmpA* gene. | (Lin et al., 2010a) |
|  | Mycotic aneurysm | Unknown | K5; *rmpA*, *icu* | Case report | (Chen et al., 2009) |
|  | 50 nonrepeat *K. pneumoniae* isolates recovered from patients with primary liver abscesses | Unknown | *K. pneumoniae* isolates with hypermucoviscosity phenotype as well as presence of *rmpA* and *iucABCD*-*iutA* genes exhibited high virulence for mouse lethality, regardless of any capsular type. | The prevalence of hypermucoviscosity phenotype, plasmid-born *rmpA*, *iucABCD*-*iutA*, *kfu*, and *allS* genes revealed 96%, 100%, 100%, 100%, and 100% in 26 capsular K1 isolates; 90%, 100%, 100%, 0%, and 0% in 10 K2 isolates; and 79%, 86%, 93%, 50%, and 0% in 14 non-K1/K2 isolates; respectively. | (Yu et al., 2008) |
|  | Community-acquired extrahepatic abscess | Unknown | K1, K2; All of the *K. pneumoniae* strains carried *rmpA*. | 88.8% of *K. pneumoniae* isolates showed the hypermucoviscosity phenotype. | (Ku et al., 2008) |
|  | Bacteremia | Unknown | Strains carrying *rmpA* were significantly associated with the hypermucoviscosity phenotype. | The prevalences of hypermucoviscosity, *rmpA*, and *magA* were 38%, 48%, and 17% in 151 *K. pneumoniae* isolates, respectively. | (Yu et al., 2006) |
|  | Community-acquired bacteremia | Unknown | The prevalence rate of *magA* among hvKP strains was 24.1%. | 41.5% of community-acquired *K. pneumoniae* bacteremia were caused by hvKP strains. | (Lee et al., 2006) |
| Singapore and Taiwan | Liver abscess | Unknown | K1, K2; Serotype K1 or K2 isolates demonstrated significantly more phagocytic resistance and virulence than did *rmpA*-positive and -negative groups of non-K1/K2 isolates. | Unknown | (Yeh et al., 2007) |
| Hong Kong, Singapore, and Taiwan | Serotype K2 isolates from three different regions in Asia | ST65, ST66, ST86, ST373, ST374, ST375, ST380, and ST434; There were two major MLST groups, ST-65-like (42%) and ST86-like (46%). | K2; All isolates contained *rmpA*. The prevalence of *iucABCD*-*iutA* gene were 25/26 (96%). | Unknown | (Lin et al., 2014) |
|  | Liver abscess | ST23 | K1 | *rmpA* and *iucABCD*-*iutA* | (Siu et al., 2011) |
| Denmark | Pyogenic liver abscess | ST23 | K1; *rmpA*, *iucABCD*-*iutA* | Case report | (Gundestrup et al., 2014) |
|  | Necrotizing fasciitis | Unknown | K2; *rmpA*, *iucABCD*-*iutA* | Case report | (Gunnarsson et al., 2009) |
| France | Seven septic localizations including bilateral diffuse cerebral abscesses | ST380 | K2; *rmpA*, *entB*, *iutA*, *ybtS* | Case report | (Hentzien et al., 2017) |
|  | Pneumonia | Unknown | *rmpA* | Case report | (Mazloum et al., 2016) |
|  | Liver abscess | ST57; CC23-KI | Unknown | Case report | (Merlet et al., 2012) |
|  | Bacteremia, pneumonia, liver abscess | ST23, ST86, ST380 | Two clones (ST86 and ST380) of serotype K2 caused five rapidly fatal bacteremia cases, whereas seven liver abscess cases were caused by K1 strains of ST23; All isolates recovered from patients with liver abscess were positive for the presence of *magA* and *rmpA* genes. | Case report | (Decre et al., 2011) |
| Germany | liver abscess | ST2398 | K2 | Case report | (Pichler et al., 2017) |
| Italy | Liver abscess | ST1861 | K1; *magA*, *rmpA*, *rmpA2*, *iucA*, *entB*, *iroB*, *ybtS* | Case report | (Arena et al., 2016) |
| Norway | Liver abscess | ST23 | K1; *magA*, *rmpA*, *iucABCD*-*iutA* | Case report | (Holmas et al., 2014) |
| Spain | Bacteremia, pyogenic liver abscesses, and pneumonia | ST23, ST380, ST86, ST23, ST65, ST25, and ST493 | K1 and K2; Among hvKP isolates, 30.2% were *magA*^+^/*rmpA*^+^, 22.6% were *magA*^-^/*rmpA*^+^, and the remaining 47.2% were *magA*^+^/*rmpA*^-^; All *magA*^+^/*rmpA*^+^ isolates were serotype K1 and ST23. | 5.4% of *K. pneumoniae* invasive isolates showed a hypermucoviscous phenotype. | (Cubero et al., 2016) |
| Sweden | Liver abscess | Unknown | K1; *rmpA*, *iucABCD*-*iutA* | Case report | (Sobirk et al., 2010) |
| Argentina | Liver abscess syndrome | ST23 | K1; *rmpA*, *magA*, *iucABCD*-*iutA* | Case report | (Vila et al., 2011) |
| Brazil | Clinical *K. pneumoniae* isolates | Unknown | K5 | The hypermucoviscous phenotype was observed in one of the *K. pneumoniae* isolates (6.7%). | (Pereira and Vanetti, 2015) |
|  | Brain and multiple liver abscesses | ST23 | K1; *magA*, *rmpA* | Case report | (Coutinho et al., 2014) |
| Canada | Community-acquired bacteremia | Unknown | K2; *rmpA* | The hypermucoviscous phenotype was present in 8.2% of *K. pneumoniae* isolates, and was associated with *rmpA* and the K2 serotype. | (Peirano et al., 2013) |
| Canada  Mexico | Pyogenic liver abscess | Unknown | K1 | Case report | (Keynan et al., 2007) |
|  | Liver abscess, septic shock, bacteremia, and pneumonia | Unknown | Unknown | Case report | (Carrillo Esper et al., 2013) |
| The United States | Osteomyelitis | ST23 | K1; *rmpA*, *rmpA2*, *magA* | Case report | (Prokesch et al., 2016) |
| The United States | Bloodstream isolates | Unknown | *rmpA*, *magA* | The hospital prevalence of isolates carrying at least one hvKP-associated gene (*rmpA* or *magA*) is 6.3 %. | (Chou et al., 2016) |
|  | Liver abscess | Unknown | K1 and K2 | Case report | (Kazanji et al., 2016) |
|  | Neck abscess, necrotizing fasciitis | Unknown | Unknown | Case report | (Ng and Frazee, 2015) |
|  | Liver abscess, osteomyelitis | Unknown | *rmpA*, *iucA*, *iro* | Case report | (Patel et al., 2014) |
|  | Liver abscess, endophthalmitis | Unknown | K1; *rmpA*, *magA* | Case report | (Sachdev et al., 2013) |
|  | Pyogenic liver abscess, bacteremia, and meningitis | Unknown | *rmpA* | Case report | (Patel et al., 2013) |
|  | Liver abscess | Unknown | K2; *rmpA* | Case report | (Pomakova et al., 2012) |
|  | Liver abscess | Unknown | K1; *rmpA* | Case report | (Fierer et al., 2011) |
|  | Liver abscess | Unknown | K2; *rmpA* | Case report | (Rivero et al., 2010) |
|  | Liver abscess, neck abscess, pyelonephritis, cholecystitis | Unknown | *rmpA*, *magA* | Case report | (Frazee et al., 2009) |
|  | Bartholin's abscess | Unknown | K2; *rmpA* | Case report | (Pinsky et al., 2009) |
|  | liver and brain abscess |  | K2 | Case report | (Doud et al., 2009) |
|  | Liver abscess | Unknown | *rmpA*, *magA* | Case report | (Nadasy et al., 2007) |
|  | Liver abscess | Unknown | *magA* | Case report | (Fang et al., 2005) |
| Algeria | 54 *K. pneumoniae* isolates from different clinical specimens | Unknown | Unknown | 9.2% of isolates were hvKP. | (El Fertas-Aissani et al., 2013) |
| Australia | Liver abscess | Unknown | K1 and K2; *rmpA*, *magA* | Unknown | (Chang et al., 2013) |
| Guadeloupe | Meningitis | CG86 | K2; *rmpA*, *iucA*, *iroN* | Case report | (Melot et al., 2016) |
| The UK, Hong Kong, Israel, Taiwan and Australia | Liver abscesses | ST23 | *rmpA* | Unknown | (Turton et al., 2007) |

*entB*, iron siderophore enterobactin; ESBL, extended-spectrum-β-lactamase; hvKP, hypervirulent *Klebsiella pneumoniae*; *iro*, iron siderophore salmochelin; *iucABCD*-*iutA*, hydroxamate iron siderophore aerobactin; *magA*, mucoviscosity-associated gene A; *pks*, colibactin encoding a polyketide-peptide genotoxin that causes genomic instability in eukaryotic cells; *rmpA* and *rmpA2*, regulator of mucoid phenotype A; UTI, Urinary tract infection; VAP, ventilator-associated pneumonia; *ybtS*, iron siderophore yersiniabactin.

**References**

Arena, F., Spanu, T., Henrici De Angelis, L., Liotti, F. M., D'Andrea, M. M., Menchinelli, G., et al. (2016) First case of bacteremic liver abscess caused by an ST260-related (ST1861), hypervirulent *Klebsiella pneumoniae*. *J Infect*. 73, 88-91. doi: 10.1016/j.jinf.2016.04.006

Carrillo Esper, R., Soto Hernandez, J. L., Pena Perez, C. A., Carrillo Cordova, L. D., Carrillo Cordova, C. A., and Carrillo Cordova, D. M. (2013) Liver abscess syndrome with lung involvement secondary to hypermucoviscosity *Klebsiella pneumoniae*. *Gac Med Mex*. 149, 102-107. doi:

Chang, L., Bastian, I., and Warner, M. (2013) Survey of *Klebsiella pneumoniae* bacteraemia in two South Australian hospitals and detection of hypermucoviscous phenotype and *magA*/*rmpA* genotypes in *K . pneumoniae* isolates. *Infection*. 41, 559-563. doi: 10.1007/s15010-012-0374-y

Chen, Y. J., Chen, S. Y., Wang, J. T., and Hsueh, P. R. (2009) Mycotic aneurysm caused by gas-forming serotype K5 *Klebsiella pneumoniae*. *Int J Infect Dis*. 13, e47-48. doi: 10.1016/j.ijid.2008.06.008

Chen, Y. T., Lai, Y. C., Tan, M. C., Hsieh, L. Y., Wang, J. T., Shiau, Y. R., et al. (2017) Prevalence and characteristics of *pks* genotoxin gene cluster-positive clinical *Klebsiella pneumoniae* isolates in Taiwan. *Sci Rep*. 7, 43120. doi: 10.1038/srep43120

Chou, A., Nuila, R. E., Franco, L. M., Stager, C. E., Atmar, R. L., and Zechiedrich, L. (2016) Prevalence of hypervirulent *Klebsiella pneumoniae*-associated genes *rmpA* and *magA* in two tertiary hospitals in Houston, TX, USA. *J Med Microbiol*. 65, 1047-1048. doi: 10.1099/jmm.0.000309

Chuang, Y. C., Lee, M. F., and Yu, W. L. (2013) Mycotic aneurysm caused by hypermucoviscous *Klebsiella pneumoniae* serotype K54 with sequence type 29: an emerging threat. *Infection*. 41, 1041-1044. doi: 10.1007/s15010-013-0447-6

Coutinho, R. L., Visconde, M. F., Descio, F. J., Nicoletti, A. G., Pinto, F. C., Silva, A. C., et al. (2014) Community-acquired invasive liver abscess syndrome caused by a K1 serotype *Klebsiella pneumoniae* isolate in Brazil: a case report of hypervirulent ST23. *Mem Inst Oswaldo Cruz*. 109, 970-971. doi:

Cubero, M., Grau, I., Tubau, F., Pallares, R., Dominguez, M. A., Linares, J., et al. (2016) Hypervirulent *Klebsiella pneumoniae* clones causing bacteraemia in adults in a teaching hospital in Barcelona, Spain (2007-2013). *Clin Microbiol Infect*. 22, 154-160. doi: 10.1016/j.cmi.2015.09.025

Decre, D., Verdet, C., Emirian, A., Le Gourrierec, T., Petit, J. C., Offenstadt, G., et al. (2011) Emerging severe and fatal infections due to *Klebsiella pneumoniae* in two university hospitals in France. *J Clin Microbiol*. 49, 3012-3014. doi: 10.1128/JCM.00676-11

Doud, M. S., Grimes-Zeppegno, R., Molina, E., Miller, N., Balachandar, D., Schneper, L., et al. (2009) A *k2A*-positive *Klebsiella pneumoniae* causes liver and brain abscess in a Saint Kitt's man. *Int J Med Sci*. 6, 301-304. doi:

El Fertas-Aissani, R., Messai, Y., Alouache, S., and Bakour, R. (2013) Virulence profiles and antibiotic susceptibility patterns of *Klebsiella pneumoniae* strains isolated from different clinical specimens. *Pathol Biol (Paris)*. 61, 209-216. doi: 10.1016/j.patbio.2012.10.004

Enani, M. A., and El-Khizzi, N. A. (2012) Community acquired *Klebsiella pneumoniae*, K1 serotype. Invasive liver abscess with bacteremia and endophthalmitis. *Saudi Med J*. 33, 782-786. doi:

Fang, F. C., Sandler, N., and Libby, S. J. (2005) Liver abscess caused by *magA*^+^ *Klebsiella pneumoniae* in North America. *J Clin Microbiol*. 43, 991-992. doi: 10.1128/JCM.43.2.991-992.2005

Fierer, J., Walls, L., and Chu, P. (2011) Recurring *Klebsiella pneumoniae* pyogenic liver abscesses in a resident of San Diego, California, due to a K1 strain carrying the virulence plasmid. *J Clin Microbiol*. 49, 4371-4373. doi: 10.1128/JCM.05658-11

Frazee, B. W., Hansen, S., and Lambert, L. (2009) Invasive infection with hypermucoviscous *Klebsiella pneumoniae*: multiple cases presenting to a single emergency department in the United States. *Ann Emerg Med*. 53, 639-642. doi: 10.1016/j.annemergmed.2008.11.007

Gundestrup, S., Struve, C., Stahlhut, S. G., and Hansen, D. S. (2014) First case of liver abscess in Scandinavia due to the international hypervirulent *Klebsiella pneumoniae* clone ST23. *Open Microbiol J*. 8, 22-24. doi: 10.2174/1874285801408010022

Gunnarsson, G. L., Brandt, P. B., Gad, D., Struve, C., and Justesen, U. S. (2009) Monomicrobial necrotizing fasciitis in a white male caused by hypermucoviscous *Klebsiella pneumoniae*. *J Med Microbiol*. 58, 1519-1521. doi: 10.1099/jmm.0.011064-0

Guo, S., Xu, J., Wei, Y., Xu, J., Li, Y., and Xue, R. (2016) Clinical and molecular characteristics of *Klebsiella pneumoniae* ventilator-associated pneumonia in mainland China. *BMC Infect Dis*. 16, 608. doi: 10.1186/s12879-016-1942-z

Guo, Y., Wang, S., Zhan, L., Jin, Y., Duan, J., Hao, Z., et al. (2017) Microbiological and clinical characteristics of hypermucoviscous *Klebsiella pneumoniae* isolates associated with invasive infections in China. *Front Cell Infect Microbiol*. 7, 24. doi: 10.3389/fcimb.2017.00024

Hentzien, M., Rosman, J., Decre, D., Brenkle, K., Mendes-Martins, L., and Mateu, P. (2017) Seven hypervirulent ST380 *Klebsiella pneumoniae* septic localizations. *Med Mal Infect*. 47, 171-173. doi: 10.1016/j.medmal.2016.10.002

Holmas, K., Fostervold, A., Stahlhut, S. G., Struve, C., and Holter, J. C. (2014) Emerging K1 serotype *Klebsiella pneumoniae* primary liver abscess: three cases presenting to a single university hospital in Norway. *Clin Case Rep*. 2, 122-127. doi: 10.1002/ccr3.77

Hyun, J. I., Kim, Y. J., Jeon, Y. H., Kim, S. I., Park, Y. J., Kang, M. W., et al. (2014) A case of ventriculitis associated with renal abscess caused by serotype K1 *Klebsiella pneumoniae*. *Infect Chemother*. 46, 120-124. doi: 10.3947/ic.2014.46.2.120

Iwasaki, Y., Inokuchi, R., Harada, S., Aoki, K., Ishii, Y., and Shinohara, K. (2017) Bacterial meningitis caused by hypervirulent *Klebsiella pneumoniae* capsular genotype K54 with development of granuloma-like nodal enhancement in the brain during the subacute phase. *Intern Med*. 56, 373-376. doi: 10.2169/internalmedicine.56.7384

Jung, S. W., Chae, H. J., Park, Y. J., Yu, J. K., Kim, S. Y., Lee, H. K., et al. (2013) Microbiological and clinical characteristics of bacteraemia caused by the hypermucoviscosity phenotype of *Klebsiella pneumoniae* in Korea. *Epidemiol Infect*. 141, 334-340. doi: 10.1017/S0950268812000933

Kazanji, N., Klein, R. E., Lohani, S., Mertens, A. N., and Le, J. (2016) A case of hypermucoviscous *Klebsiella pneumoniae* liver abscess syndrome in an Iraqi male. *QJM*. 109, 493-494. doi: 10.1093/qjmed/hcw049

Keynan, Y., Karlowsky, J. A., Walus, T., and Rubinstein, E. (2007) Pyogenic liver abscess caused by hypermucoviscous *Klebsiella pneumoniae*. *Scand J Infect Dis*. 39, 828-830. doi: 10.1080/00365540701266763

Kim, Y. J., Kim, S. I., Kim, Y. R., Wie, S. H., Lee, H. K., Kim, S. Y., et al. (2017) Virulence factors and clinical patterns of hypermucoviscous *Klebsiella pneumoniae* isolated from urine. *Infect Dis (Lond)*. 49, 178-184. doi: 10.1080/23744235.2016.1244611

Kishibe, S., Okubo, Y., Morino, S., Hirotaki, S., Tame, T., Aoki, K., et al. (2016) Pediatric hypervirulent *Klebsiella pneumoniae* septic arthritis. *Pediatr Int*. 58, 382-385. doi: 10.1111/ped.12806

Ku, Y. H., Chuang, Y. C., and Yu, W. L. (2008) Clinical spectrum and molecular characteristics of *Klebsiella pneumoniae* causing community-acquired extrahepatic abscess. *J Microbiol Immunol Infect*. 41, 311-317. doi:

Lee, H. C., Chuang, Y. C., Yu, W. L., Lee, N. Y., Chang, C. M., Ko, N. Y., et al. (2006) Clinical implications of hypermucoviscosity phenotype in *Klebsiella pneumoniae* isolates: association with invasive syndrome in patients with community-acquired bacteraemia. *J Intern Med*. 259, 606-614. doi: 10.1111/j.1365-2796.2006.01641.x

Lee, I. R., Molton, J. S., Wyres, K. L., Gorrie, C., Wong, J., Hoh, C. H., et al. (2016) Differential host susceptibility and bacterial virulence factors driving *Klebsiella* liver abscess in an ethnically diverse population. *Sci Rep*. 6, 29316. doi: 10.1038/srep29316

Lin, J. C., Koh, T. H., Lee, N., Fung, C. P., Chang, F. Y., Tsai, Y. K., et al. (2014) Genotypes and virulence in serotype K2 *Klebsiella pneumoniae* from liver abscess and non-infectious carriers in Hong Kong, Singapore and Taiwan. *Gut Pathog*. 6, 21. doi: 10.1186/1757-4749-6-21

Lin, W. H., Wang, M. C., Tseng, C. C., Ko, W. C., Wu, A. B., Zheng, P. X., et al. (2010a) Clinical and microbiological characteristics of *Klebsiella pneumoniae* isolates causing community-acquired urinary tract infections. *Infection*. 38, 459-464. doi: 10.1007/s15010-010-0049-5

Lin, Y. T., Jeng, Y. Y., Chen, T. L., and Fung, C. P. (2010b) Bacteremic community-acquired pneumonia due to *Klebsiella pneumoniae*: clinical and microbiological characteristics in Taiwan, 2001-2008. *BMC Infect Dis*. 10, 307. doi: 10.1186/1471-2334-10-307

Luo, Y., Wang, Y., Ye, L., and Yang, J. (2014) Molecular epidemiology and virulence factors of pyogenic liver abscess causing *Klebsiella pneumoniae* in China. *Clin Microbiol Infect*. 20, O818-824. doi: 10.1111/1469-0691.12664

Mazloum, M., Le Meur, M., Barnaud, G., and Messika, J. (2016) Hypermucoviscous *Klebsiella pneumoniae* pneumonia: follow the string! *Intensive Care Med*. 42, 2092-2093. doi: 10.1007/s00134-016-4363-y

Melot, B., Brisse, S., Breurec, S., Passet, V., Malpote, E., Lamaury, I., et al. (2016) Community-acquired meningitis caused by a CG86 hypervirulent *Klebsiella pneumoniae* strain: first case report in the Caribbean. *BMC Infect Dis*. 16, 736. doi: 10.1186/s12879-016-2065-2

Merlet, A., Cazanave, C., Dutronc, H., de Barbeyrac, B., Brisse, S., and Dupon, M. (2012) Primary liver abscess due to CC23-K1 virulent clone of *Klebsiella pneumoniae* in France. *Clin Microbiol Infect*. 18, E338-339. doi: 10.1111/j.1469-0691.2012.03953.x

Mita, N., Narahara, H., Okawa, M., Hinohara, H., Kunimoto, F., Haque, A., et al. (2012) Necrotizing fasciitis following psoas muscle abscess caused by hypermucoviscous *Klebsiella pneumoniae*. *J Infect Chemother*. 18, 565-568. doi: 10.1007/s10156-011-0338-7

Nadasy, K. A., Domiati-Saad, R., and Tribble, M. A. (2007) Invasive *Klebsiella pneumoniae* syndrome in North America. *Clin Infect Dis*. 45, e25-28. doi: 10.1086/519424

Nakamoto, K., Koide, T., Nagatomo, T., Tamura, M., Higaki, M., Takata, S., et al. (2011) Severe primary liver abscess and septic pulmonary embolism due to *Klebsiella pneumoniae* with hypermucoviscosity phenotype. *Kansenshogaku Zasshi*. 85, 366-369. doi:

Namikawa, H., Yamada, K., Fujimoto, H., Oinuma, K. I., Tochino, Y., Takemoto, Y., et al. (2016) Two unusual cases of successful treatment of hypermucoviscous *Klebsiella pneumoniae* invasive syndrome. *BMC Infect Dis*. 16, 680. doi: 10.1186/s12879-016-2011-3

Ng, D., and Frazee, B. (2015) Necrotizing fasciitis caused by hypermucoviscous Klebsiella pneumoniae in a Filipino female in North America. *West J Emerg Med*. 16, 165-168. doi: 10.5811/westjem.2014.11.23599

Patel, G., Shah, N., and Sharma, R. (2013) Pyogenic Liver Abscess, Bacteremia, and Meningitis with Hypermucoviscous Klebsiella pneumoniae: An Unusual Case Report in a Human T-Cell Lymphotropic Virus Positive Patient of Caribbean Origin in the United States. *Case Rep Infect Dis*. 2013, 676340. doi: 10.1155/2013/676340

Patel, P. K., Russo, T. A., and Karchmer, A. W. (2014) Hypervirulent Klebsiella pneumoniae. *Open Forum Infect Dis*. 1, ofu028. doi: 10.1093/ofid/ofu028

Peirano, G., Pitout, J. D., Laupland, K. B., Meatherall, B., and Gregson, D. B. (2013) Population-based surveillance for hypermucoviscosity *Klebsiella pneumoniae* causing community-acquired bacteremia in Calgary, Alberta. *Can J Infect Dis Med Microbiol*. 24, e61-64. doi:

Pereira, S. C., and Vanetti, M. C. (2015) Potential virulence of *Klebsiella* sp. isolates from enteral diets. *Braz J Med Biol Res*. 48, 782-789. doi: 10.1590/1414-431X20154316

Pichler, C., Buchsel, M., Rossen, J. W., Vavra, M., Reuter, S., Kern, W. V., et al. (2017) First report of invasive liver abscess syndrome with endophthalmitis caused by a K2 serotype ST2398 hypervirulent *Klebsiella pneumoniae* in Germany, 2016. *New Microbes New Infect*. 17, 77-80. doi: 10.1016/j.nmni.2017.02.006

Pinsky, B. A., Baron, E. J., Janda, J. M., and Banaei, N. (2009) Bartholin's abscess caused by hypermucoviscous *Klebsiella pneumoniae*. *J Med Microbiol*. 58, 671-673. doi: 10.1099/jmm.0.006734-0

Pomakova, D. K., Hsiao, C. B., Beanan, J. M., Olson, R., MacDonald, U., Keynan, Y., et al. (2012) Clinical and phenotypic differences between classic and hypervirulent *Klebsiella pneumoniae*: an emerging and under-recognized pathogenic variant. *Eur J Clin Microbiol Infect Dis*. 31, 981-989. doi: 10.1007/s10096-011-1396-6

Prokesch, B. C., TeKippe, M., Kim, J., Raj, P., TeKippe, E. M., and Greenberg, D. E. (2016) Primary osteomyelitis caused by hypervirulent Klebsiella pneumoniae. *Lancet Infect Dis*. 16, e190-195. doi: 10.1016/S1473-3099(16)30021-4

Qu, T. T., Zhou, J. C., Jiang, Y., Shi, K. R., Li, B., Shen, P., et al. (2015) Clinical and microbiological characteristics of *Klebsiella pneumoniae* liver abscess in East China. *BMC Infect Dis*. 15, 161. doi: 10.1186/s12879-015-0899-7

Rivero, A., Gomez, E., Alland, D., Huang, D. B., and Chiang, T. (2010) K2 serotype *Klebsiella pneumoniae* causing a liver abscess associated with infective endocarditis. *J Clin Microbiol*. 48, 639-641. doi: 10.1128/JCM.01779-09

Sachdev, D. D., Yin, M. T., Horowitz, J. D., Mukkamala, S. K., Lee, S. E., and Ratner, A. J. (2013) *Klebsiella pneumoniae* K1 liver abscess and septic endophthalmitis in a U.S. resident. *J Clin Microbiol*. 51, 1049-1051. doi: 10.1128/JCM.02853-12

Seo, R., Kudo, D., Gu, Y., Yano, H., Aoyagi, T., Omura, T., et al. (2016) Invasive liver abscess syndrome caused by *Klebsiella pneumoniae* with definite K2 serotyping in Japan: a case report. *Surg Case Rep*. 2, 72. doi: 10.1186/s40792-016-0201-2

Siu, L. K., Fung, C. P., Chang, F. Y., Lee, N., Yeh, K. M., Koh, T. H., et al. (2011) Molecular typing and virulence analysis of serotype K1 Klebsiella pneumoniae strains isolated from liver abscess patients and stool samples from noninfectious subjects in Hong Kong, Singapore, and Taiwan. *J Clin Microbiol*. 49, 3761-3765. doi: 10.1128/JCM.00977-11

Sobirk, S. K., Struve, C., and Jacobsson, S. G. (2010) Primary *Klebsiella pneumoniae* liver abscess with metastatic spread to lung and eye, a North-European case report of an emerging syndrome. *Open Microbiol J*. 4, 5-7. doi: 10.2174/1874285801004010005

Sun, Y., Wu, H., and Shen, D. (2016) Clinical and molecular analysis of *Klebsiella pneumoniae* causing liver abscess in China. *J Mol Microbiol Biotechnol*. 26, 245-251. doi: 10.1159/000444367

Suzuki, K., Nakamura, A., Enokiya, T., Iwashita, Y., Tomatsu, E., Muraki, Y., et al. (2013) Septic arthritis subsequent to urosepsis caused by hypermucoviscous *Klebsiella pneumoniae*. *Intern Med*. 52, 1641-1645. doi:

Togawa, A., Toh, H., Onozawa, K., Yoshimura, M., Tokushige, C., Shimono, N., et al. (2015) Influence of the bacterial phenotypes on the clinical manifestations in *Klebsiella pneumoniae* bacteremia patients: a retrospective cohort study. *J Infect Chemother*. 21, 531-537. doi: 10.1016/j.jiac.2015.04.004

Turton, J. F., Englender, H., Gabriel, S. N., Turton, S. E., Kaufmann, M. E., and Pitt, T. L. (2007) Genetically similar isolates of *Klebsiella pneumoniae* serotype K1 causing liver abscesses in three continents. *J Med Microbiol*. 56, 593-597. doi: 10.1099/jmm.0.46964-0

Vila, A., Cassata, A., Pagella, H., Amadio, C., Yeh, K. M., Chang, F. Y., et al. (2011) Appearance of *Klebsiella pneumoniae* liver abscess syndrome in Argentina: case report and review of molecular mechanisms of pathogenesis. *Open Microbiol J*. 5, 107-113. doi: 10.2174/1874285801105010107

Yan, J. J., Zheng, P. X., Wang, M. C., Tsai, S. H., Wang, L. R., and Wu, J. J. (2015) Allocation of *Klebsiella pneumoniae* bloodstream isolates into four distinct groups by *ompK36* typing in a Taiwanese university hospital. *J Clin Microbiol*. 53, 3256-3263. doi: 10.1128/JCM.01152-15

Ye, M., Tu, J., Jiang, J., Bi, Y., You, W., Zhang, Y., et al. (2016) Clinical and genomic analysis of liver abscess-causing *Klebsiella pneumoniae* identifies new liver abscess-associated virulence genes. *Front Cell Infect Microbiol*. 6, 165. doi: 10.3389/fcimb.2016.00165

Yeh, K. M., Kurup, A., Siu, L. K., Koh, Y. L., Fung, C. P., Lin, J. C., et al. (2007) Capsular serotype K1 or K2, rather than *magA* and *rmpA*, is a major virulence determinant for *Klebsiella pneumoniae* liver abscess in Singapore and Taiwan. *J Clin Microbiol*. 45, 466-471. doi: 10.1128/JCM.01150-06

Yu, W. L., Ko, W. C., Cheng, K. C., Lee, H. C., Ke, D. S., Lee, C. C., et al. (2006) Association between *rmpA* and *magA* genes and clinical syndromes caused by *Klebsiella pneumoniae* in Taiwan. *Clin Infect Dis*. 42, 1351-1358. doi: 10.1086/503420

Yu, W. L., Ko, W. C., Cheng, K. C., Lee, C. C., Lai, C. C., and Chuang, Y. C. (2008) Comparison of prevalence of virulence factors for *Klebsiella pneumoniae* liver abscesses between isolates with capsular K1/K2 and non-K1/K2 serotypes. *Diagn Microbiol Infect Dis*. 62, 1-6. doi: 10.1016/j.diagmicrobio.2008.04.007

Yu, W. L., Lee, M. F., Chang, M. C., and Chuang, Y. C. (2015a) Intrapersonal mutation of *rmpA* and *rmpA2*: A reason for negative hypermucoviscosity phenotype and low virulence of *rmpA*-positive *Klebsiella pneumoniae* isolates. *J Glob Antimicrob Resist*. 3, 137-141. doi: 10.1016/j.jgar.2015.03.008

Yu, W. L., Lee, M. F., Tang, H. J., Chang, M. C., and Chuang, Y. C. (2015b) Low prevalence of rmpA and high tendency of rmpA mutation correspond to low virulence of extended spectrum beta-lactamase-producing Klebsiella pneumoniae isolates. *Virulence*. 6, 162-172. doi: 10.1080/21505594.2015.1016703

Zamani, A., Yousefi Mashouf, R., Ebrahimzadeh Namvar, A. M., and Alikhani, M. Y. (2013) Detection o*f magA* gene in *Klebsiella* spp. isolated from clinical samples. *Iran J Basic Med Sci*. 16, 173-176. doi:

Zhang, Y., Sun, J., Mi, C., Li, W., Zhao, S., Wang, Q., et al. (2015) First report of two rapid-onset fatal infections caused by a newly emerging hypervirulent *K. pneumonia* ST86 strain of serotype K2 in China. *Front Microbiol*. 6, 721. doi: 10.3389/fmicb.2015.00721

Zhao, J., Chen, J., Zhao, M., Qiu, X., Chen, X., Zhang, W., et al. (2016) Multilocus sequence types and virulence determinants of hypermucoviscosity-positive *Klebsiella pneumoniae* isolated from community-acquired infection cases in Harbin, North China. *Jpn J Infect Dis*. 69, 357-360. doi: 10.7883/yoken.JJID.2015.321
